# Vaginal Foreign Body Forgotten for 20 Years in a Postmenopausal Female: A Case Report

**DOI:** 10.7759/cureus.33132

**Published:** 2022-12-30

**Authors:** Nalini Sharma, Darshini S Vegda, Vishwa S Gandhi

**Affiliations:** 1 Obstetrics and Gynecology, Geetanjali Medical College and Hospital, Udaipur, IND

**Keywords:** recurrent urinary infection, foul-smelling vaginal discharge, urinary bladder perforation, cervical cancer, vesicovaginal fistula, quacks, postmenopausal female, prolapse treatment, forgotten, vaginal foreign body

## Abstract

Here, we present a case of a 70-year-old female, menopausal for 20 years, who came to our outpatient department with complaints of recurrent urinary tract infection (UTI) and foul-smelling vaginal discharge. She also had occasional abdominal pain and experienced social stigma due to the foul smell. No history of fever or bleeding per vaginum and no obvious vulval/cervicovaginal growth or uterovaginal prolapse was found. Per speculum and per vaginal examinations were carried out. On examination, a foreign body of approximately 10 × 10 cm was found free in the atrophic vaginal canal. She gave a history of insertion 20 years back, as treatment of prolapse, by some quack. The foreign body was removed, and no trauma occurred during the process of removal. She was then managed conservatively with antibiotics and supportive treatment. In the modern era, instead of ring pessary, the use of plastic balls or some fruit as a non-pharmacologic treatment of prolapse is unheard of. Our case draws attention that every gynecologist should be aware of the entity and have knowledge about its extraction, to give better care to menopausal females.

## Introduction

A vaginal foreign body (VFB) in a postmenopausal female is a rare occurrence in the modern era. They cause symptoms such as foul-smelling vaginal discharge, itching on the vulva, bleeding per vaginum, or recurrent urinary tract infections (UTIs). Vesicovaginal fistulas, rectovaginal fistulas, bladder injury, and multiple surgeries can be grave consequences. Only three instances of foreign body placement in the vagina that were retained for an extended period have been reported [1-3]. Ours is the fourth reported case of a forgotten vaginal foreign body in a postmenopausal female. This report describes the challenges of the diagnosis and management of foreign bodies.

## Case presentation

A 70-year-old postmenopausal woman came to our outpatient department complaining of foul-smelling vaginal discharge, recurring urinary tract infection, occasional abdominal pain, and social stigma resulting from her foul odor. No other symptoms were reported, such as fever, vaginal bleeding, vulval/cervicovaginal growth, urinary dribbling, or uterovaginal prolapse. She had six previous normal deliveries. She was in good physical and mental health during the examination. She was not sexually active during this period. Based on history, a presumptive diagnosis of urinary tract infection, atrophic vaginitis, or carcinoma cervix was made.

**Figure 1:**
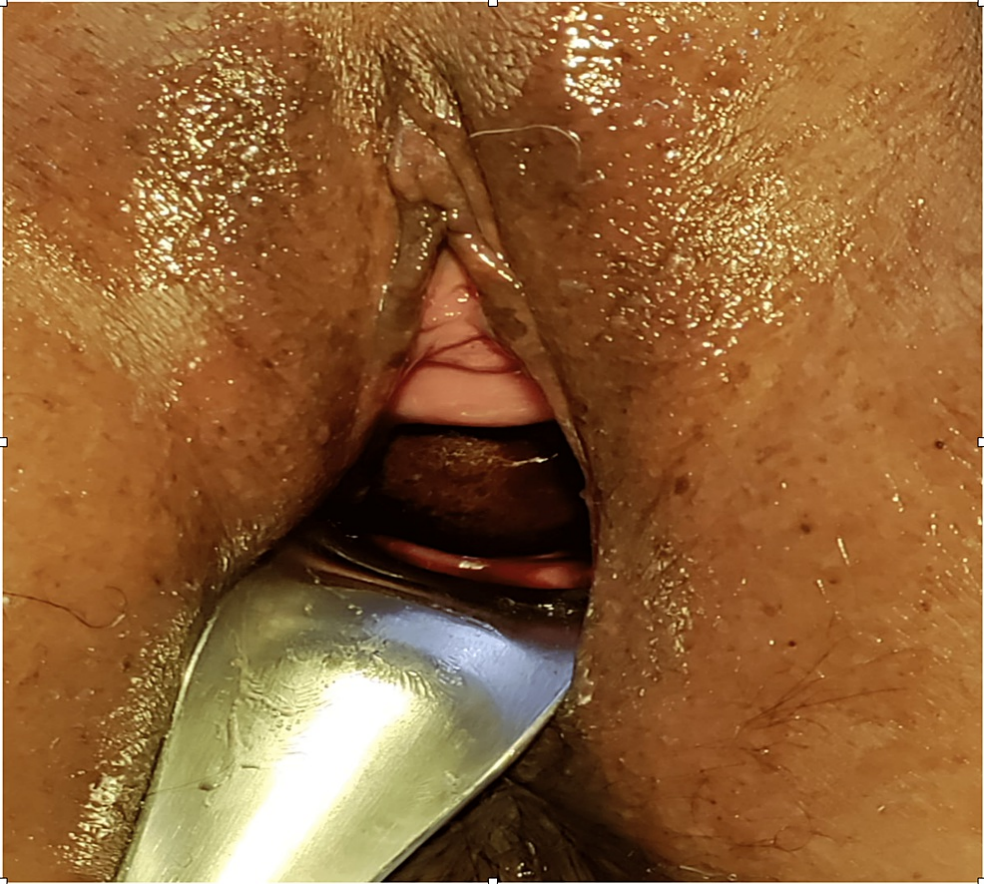
Per speculum examination

On inspection, putrid and foul-smelling vaginal discharge was present. No other finding was present. During a per vaginal examination, the cervix was not felt. A large intravaginal foreign body measuring approximately 10 × 10 cm obscured the cervix. (Figure 1). It was round, brownish in color, and woody hard and felt hollow inside due to its lightweight, mobility, and odorousness. The vaginal canal distal to the foreign body had become friable and atrophied, causing narrowing. The introitus was narrow too. Per rectal examination was done, and it was found that rectal mucosa was free with no growth felt either in the vagina or rectum. A small plastic ball or wood apple was identified as the potential cause.

At this point, the patient provided a history of a foreign body being inserted by a dai approximately 20 years ago. It was inserted to treat prolapse.

She was admitted to the hospital and underwent routine blood tests (viz., hemogram, renal function test, liver function test, and viral markers). All blood tests were within normal limits. Pap smear could not be done at this point.

Under sedation, Allis forceps was used to remove the foreign body. It was unsuccessful as Allis forceps could not hold the foreign body stoutly between its two jaws. The ventouse was used, but the foreign body failed to be removed. Suction could not form between the foreign body and the silicone cup of the ventouse. Wrigley’s forceps were then employed, but it was futile too. The foreign body kept rotating between the two blades and the distal vaginal canal, and the introitus was too narrow for forceps to come out. An episiotomy felt necessary. With the possibility of episiotomy in mind, a final attempt was made with vulsellum. Because it is a strong instrument with teeth, the vulsellum proved to be the right choice. It had a firm grip on the foreign body. As surgeons were trying to pull it out, the foreign body broke.

**Figure 2:**
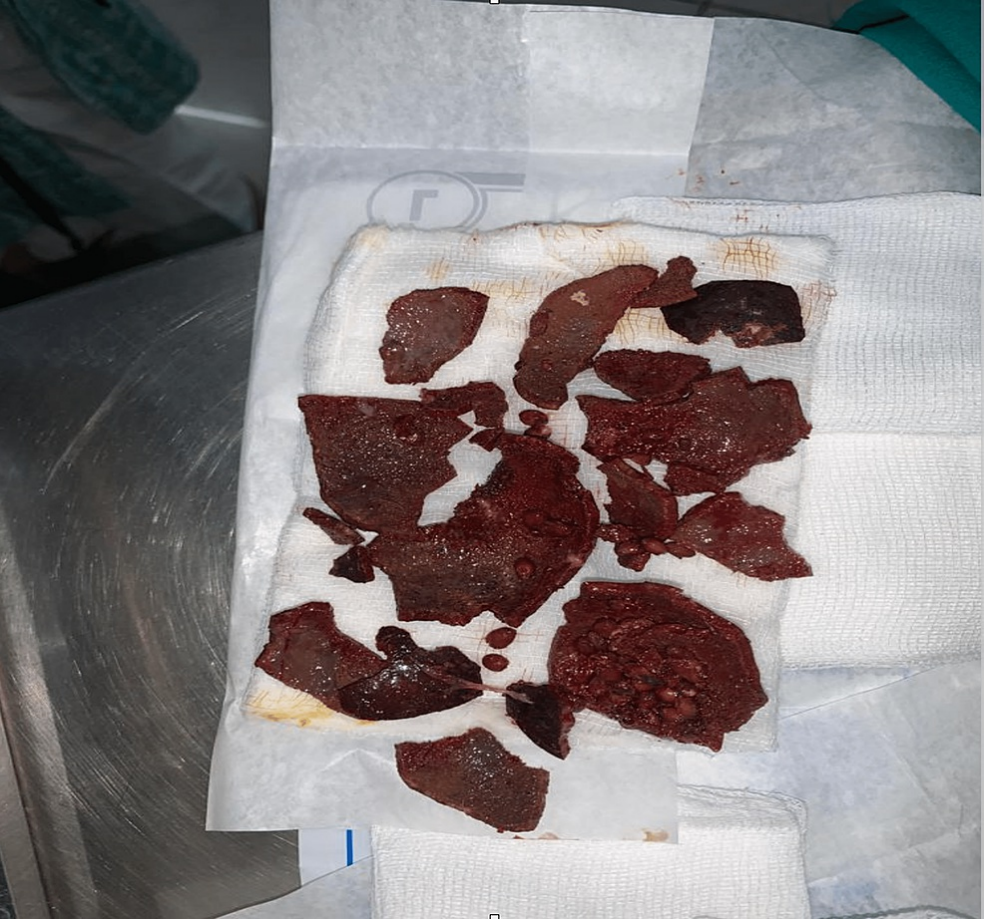
Wood apple (broken) after extraction

It was confirmed to be a wood apple as the wall of the foreign body was 3-4 mm thick and had numerous seeds inside. The odor was unbearably offensive once it broke. The pieces of the disintegrated foreign body were sharp and jagged. They posed a significant risk of tearing through the friable and atrophied vaginal wall, causing damage to adjacent organs if removed inefficiently. So, it was removed piece by piece using vulsellum with great caution (Figure 2).

A thorough vaginal lavage was done using normal saline and 5% betadine solution. No trauma occurred during the process of the removal of the foreign body. Parenteral antibiotics and supportive treatment were administered. The results of her blood investigations were within normal limits. USG was done after the removal of the foreign body. Neither any injuries nor any intravaginal retained products were seen on ultrasound. Pap smear was done. It was reported as a grade II inflammatory smear. Intravenous metronidazole and amoxicillin + clavulanic acid were administered, followed by an oral antibiotic course for a total of 14 days. The patient was discharged in stable condition.

Follow-up was done on the seventh day and after one month. On both occasions, per speculum examination showed normal findings, and the cervix was visualized. The cervix had no growth but was flushed with the vagina. Fornices were blunt. There was no evidence of cystocele, urethrocele, or rectocele. The vaginal wall healed well. There were no urinary complaints. Repeat Pap smear was done, and the report came within normal limits.

## Discussion

The vaginal canal is utilized to implant contraceptives, suppositories to induce abortion or antibiotics and prebiotics, and ring pessary for urogenital prolapse. All other insertions, whether herbs for illegal abortion/inducing labor, toys for sexual gratification, and objects for sexual abuse and child abuse or by psychiatric females, are labeled “foreign bodies” [2].

Vaginal foreign bodies (VFBs) recovered from the children and adolescent age group are either used for child abuse/sexual abuse or sexual gratification. In the reproductive age group, sexual violence or abuse stands out as a common cause [4,5].

The complaints are determined by the VFB’s size, shape, composition, and duration since insertion. Common ailments include recurrent urinary tract infections, urinary leakage, vaginal bleeding, abdominal pain, and purulent or foul-smelling discharge, with or without blood stains. Unattended VFBs have also presented as vesicovaginal, urethrovaginal, rectovaginal fistulas, vaginolith, and vesical calculus. They present with severe consequences such as vesicovaginal, urethrovaginal, rectovaginal fistulas, and pyuria, requiring surgery, sometimes even in multiple sittings. Difficult extraction may result in injury to the adjacent organs, as reported by Puppo et al. [1]. Children with VFBs do require psychological consults, as the cause is more than often sexual abuse [5,6].

Ring pessaries, both neglected and well-cared for, used for nonsurgical correction of urogenital prolapse have been shown to cause morbidity [7,8]. Strangely, in this case, the wood apple implanted 20 years ago did not result in severe morbidity in the patient. It neither eroded the vaginal wall resulting in a fistula nor caused any adhesions.

Most case reports have adolescent girls or females in the reproductive age group (<25 years old). Puppo et al., Chopra et al., and Ciebiera et al. reported cases of females aged 74, 50, and 73 years, respectively [1-3]. All of them had foreign bodies retained for a long duration.

A variety of foreign bodies have been extracted from the vagina. They differ depending on the age of the female and the purpose of ingress. The commonly removed foreign bodies from the vagina of children, adolescents, or females in the reproductive age group include hairpins, buttons, seeds, toy parts, objects used in foreplay, pen caps, bottle caps, toilet paper, and illegal drugs for trafficking [1,3]. In the perimenopausal or postmenopausal age group of females, plastic bottle caps, hexagonal plastic pieces, and objects to treat prolapses, such as ring pessary, wood apples, and plastic balls, are reported [7,8].

Diagnosis is more straightforward and quicker if one is aware of the entity of vaginal foreign bodies; otherwise, it is usually delayed [9]. A vaginal examination usually makes the diagnosis of large foreign objects. Smaller objects require USG of the pelvis, X-ray of the pelvis, magnetic resonance imaging, and a strong suspicion of foreign bodies. As was the case in all three studies previously cited [1-3] and ours, diagnosis is delayed as help is sought from other specialties for complaints such as urinary tract infections, abdominal pain, or fever. Differential diagnoses made to reach the current one had been genital infections, atrophic vaginitis, and cervical carcinoma.

Denial is common in cases where vaginal foreign bodies have been retained. The fear of embarrassment or humiliation results in delay in reaching the caregiver and, subsequently, grave consequences for the female.

## Conclusions

Vaginal foreign bodies in postmenopausal females are uncommon in today’s world. It is challenging to diagnose it accurately in time. As the saying goes, “our eyes see what our minds know.” Any female who presents with recurrent urinary tract infections and vaginal discharge must be examined by a gynecologist. Every gynecologist should be aware of the entity and have a basic understanding to extract them. This could reduce morbidity associated with rare cases of forgotten intravaginal foreign bodies.
